# Laser Acupuncture Exerts Neuroprotective Effects via Regulation of* Creb*,* Bdnf*,* Bcl-2*, and* Bax* Gene Expressions in the Hippocampus

**DOI:** 10.1155/2017/7181637

**Published:** 2017-03-20

**Authors:** Yeong-Chan Yun, Dongyeop Jang, Sun-Bee Yoon, Dohyeong Kim, Dong-Hee Choi, O-Sang Kwon, Yu-Mi Lee, Daehwan Youn

**Affiliations:** ^1^Department of Meridian and Acupuncture Point, College of Korean Medicine, Dongshin University, 185, Geonjae-ro, Naju, Republic of Korea; ^2^Acupuncture, Moxibustion, and Acupuncture Point Research Group, Korea Institute of Oriental Medicine, 1672, Yuseong-daero, Yuseong-gu, Daejeon, Republic of Korea

## Abstract

Acupuncture has a positive effect on cognitive deficits. However, the effects of laser acupuncture (LA) on cognitive function and its mechanisms of action are unclear. The present study aimed to evaluate the effects of LA on middle cerebral artery occlusion- (MCAO-) induced cognitive impairment and its mechanisms of action. Transient focal cerebral ischemia was modeled in adult Sprague-Dawley rats by MCAO. After LA or manual-acupuncture (MA) treatment at the GV20 and HT7 for 2 weeks, hippocampal-dependent memory was evaluated using the Morris water maze (MWM) test. The hippocampus was dissected to analyze choline acetyltransferase (ChAT) immunoreactivity and* Creb*,* Bdnf*,* Bcl-2*, and* Bax* gene expressions. MWM test demonstrated a significant improvement in hippocampal-dependent memory in the MCAO rats after LA treatment. LA treatment significantly reversed the postischemic decrease in ChAT immunoreactivity in the hippocampal CA1 region. LA treatment significantly normalized gene expression in the hippocampus which had been altered by MCAO, especially upregulating gene expression of Creb, Bdnf, and Bcl-2 and downregulating gene expression of Bax. This study suggests that LA treatment could improve cognitive impairment in MCAO rats to enhance the cholinergic system in the hippocampal CA1 region and to exert a neuroprotective effect by regulating* Creb*,* Bdnf*,* Bcl-2*, and* Bax* gene expressions.

## 1. Introduction

In Asian countries, such as China, Korea, Japan, and Vietnam, acupuncture has been one of the most widely used treatment methods in traditional medicine for thousands of years [1]. In complementary and alternative medicine, it is frequently utilized for neurological disorders, such as stroke and dementia, because of its neuroprotective effects and its ability to improve poor cognitive function [2, 3]. In addition to conventional manual acupuncture (MA), which only uses needles, other acupuncture approaches, such as electrostimulation, have been reported [4, 5]. One of the alternative techniques in acupuncture is laser acupuncture (LA), which utilizes laser irradiation. LA treatment involves stimulation of the appropriate acupuncture points based on acupuncture theory, using a low-intensity, nonthermal laser [6]. Several studies have reported that LA treatment alleviates cognitive deficits in animal models through various mechanisms, such as reducing oxidative stress and protecting against damage to cholinergic and dopaminergic neurons [7–9].

Cerebrovascular disease is the second most common cause of acquired cognitive impairment and dementia and has been reported to contribute to impaired cognition in neurodegenerative dementia [10]. The hippocampus is one of the most important regions of the brain with respect to cognition, learning, and memory, and the hippocampal CA1 region has been shown to be particularly vulnerable to ischemic insult [11]. In previous studies, after induction of focal cerebral ischemia by middle cerebral artery occlusion (MCAO), neurodegeneration was reported in the hippocampal CA1 region, accompanied by long-term cognitive deficits [12, 13]. Neuron survival after such ischemic brain injury has been shown to be mediated by regulation of cAMP response element- (CRE-) mediated gene expressions, including cAMP response binding protein* (Creb)* [14], brain-derived neurotrophic factor* (Bdnf)* [15], and B-cell lymphoma 2* (Bcl-2)* [16] expressions.

In our previous study, when LA treatment was applied to two acupuncture points (HT9 and LR1) in MCAO rats, an antiapoptotic effect was observed, with counterregulation of* Bcl-2* and Bcl-2-associated X protein* (Bax)* gene expressions [17]. Several previous studies have reported that acupuncture stimulation at the acupuncture points GV20 [18] and HT7 [19] in animal models showed potential neuroprotective effects and improvement of cognitive deficits. However, the molecular mechanisms associated with LA's neuroprotective effects and alleviation of cognitive deficits after brain ischemia are unclear. Therefore, the present study aimed to evaluate the effects of LA at the acupuncture points GV20 and HT7 on MCAO-induced cognitive impairment and its mechanisms of action.

## 2. Materials and Methods

### 2.1. Animals and the MCAO Model

The study included 8-week-old male Sprague-Dawley (SD) rats weighing 260–300 g. The rats were acclimated in a temperature-controlled (22 ± 3°C) environment with a constant 12-hour light/dark cycle and ad libitum access to food and water 3 days prior to surgery. All procedures in this experiment were performed in accordance with the National Institutes of Health Guide for the Care and Use of Laboratory Animals (NIH Publications number 8023), revised in 1996, and were approved by the Institutional Animal Care and Use Committee of Dongshin University (2015-04-04). A rat model of transient focal cerebral ischemia was created by occlusion of the left middle cerebral artery, in accordance with the surgical procedure presented by Longa et al. [20]. In brief, the rats were subjected to inhalation anesthesia (following induction with 5% isoflurane, anesthesia was maintained at a concentration of 2%). An incision was made in the neck at the midline, and the left common carotid artery was exposed between the sternocleidomastoid and omohyoid muscles. The terminal branch of the left internal carotid artery (1 cm from where it branches from the left common carotid artery) was perforated with microvascular scissors, and an intraluminal filament (0.28 × 20 mm, rounded tip) covered in dental impression material (Durelon, ESPE, Seefeld, Germany) was inserted.

### 2.2. Experimental Design

In order to observe the effects of LA on cognitive impairment in the MCAO rat model, we arbitrarily divided 24 SD rats into the following four groups: the naïve, control (MCAO only), MA (MCAO + MA), and LA (MCAO + LA) groups.

### 2.3. Acupuncture Treatment

**Figure 1:**
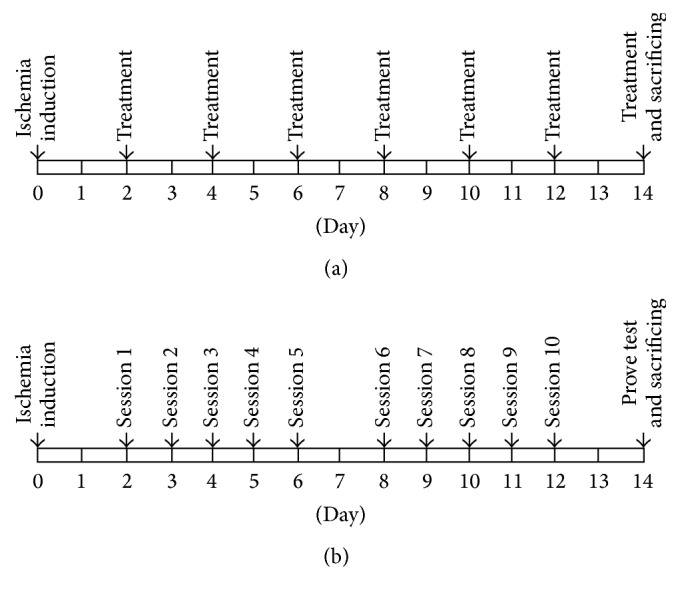
Schematic design of the experimental procedures for acupuncture treatment (a) and the Morris water maze test (b).

**Figure 2:**
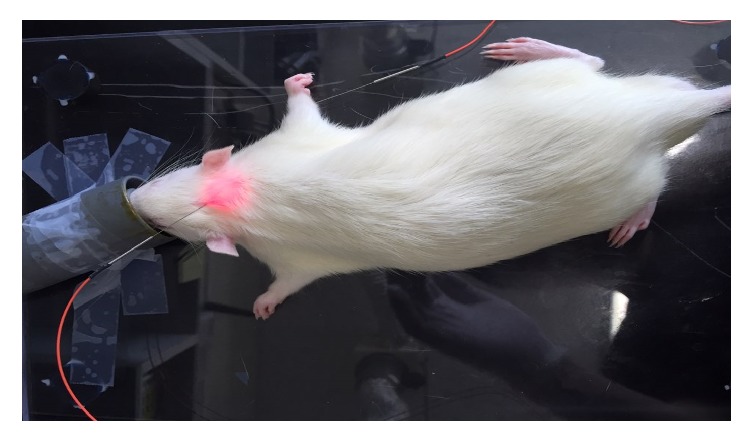
An image of laser acupuncture treatment being applied to the two acupuncture points GV20 and HT7.

After 48 postoperative hours, acupuncture treatment was performed 4 hours before the Morris water maze task in the behavioral test period. Acupuncture treatment was performed using either an invasive LA apparatus (Ellise-005, Ver. 1.0.1, Wontech, Daejeon, Korea; for the LA group; Table 1) or acupuncture needles (0.20 × 30 mm, sterilized disposable stainless steel, HLMedical, Yeoju, Korea; for the MA group). The acupuncture points Baekhoe (GV20) and Sinmun (HT7) were stimulated for 5 minutes, once every 2 days, for 2 weeks, resulting in a total of seven stimulations at each point (Figure 1(a)). HT7 was stimulated unilaterally on the right side (i.e., contralateral to the left MCAO). GV20 was anatomically located at the intersection of the line connecting the apexes of the two auricles and the median line of the head, while HT7 was located at the transverse crease of the wrist of the forepaw, radial to the tendon of the flexor carpi ulnaris muscle [21]. The LA stimulation parameters were as follows: laser-guided needles (200 *μ*min diameter, with an optic fiber 125 *μ*m in diameter); diode laser irradiation; wavelength, 650 nm; intensity, 30 mW; and repetition rate, 100 Hz. For both LA and MA, the needles were inserted at GV20 (obliquely) and HT7 (perpendicularly) to a depth of approximately 2-3 mm. The naïve and control groups only underwent 5 minutes of inhalation anesthesia. A rat undergoing LA treatment is presented in Figure 2.

### 2.4. Morris Water Maze Test

Environmental factors relevant to the Morris water maze task were based on the protocol presented by Vorhees and Williams [22]. In brief, the water maze consisted of a polypropylene circular pool (diameter, 120 cm; height, 50 cm), and the pool was divided into four identical quadrants (northeast, northwest, southeast, and southwest). A water temperature of 22 ± 1°C was maintained inside the pool, and ink was added to make the water opaque. In the Morris water maze task, a platform (diameter, 20 cm; height, 32 cm; and depth, 1-2 cm below the surface of the water) was located in the center of one of the four quadrants. The pool was surrounded by numerous external clues. A camera lens (CS Mount 1/3′′ 4 mm Fixed Focus Manual Iris Lens/T0412FICS, CBC AMERICAS, Cary, NC) was installed and fixed above the pool, and automated video tracking of the swim paths of the rats was implemented in all trials, using a tracking program (Smart ver. 3.0.01, Panlab/Harvard Apparatus, Holliston, MA) linked to the camera.

#### 2.4.1. Acquisition Trial

A hidden platform located in the northwest quadrant was the only place for the rats to avoid the water. A rat was placed arbitrarily in one of the other quadrants facing the wall of the pool. When the rat reached the platform, the timer was stopped immediately. If a rat was unable to find the platform within 60 seconds, it was guided gently to the platform, and the escape latency time was recorded as 60 seconds. When a rat climbed onto the platform, it was not removed immediately, but rather a 15-second intertrial interval was provided for the rat to remember the area around the pool. The rats were tested in sessions consisting of four trials a day, and over a 2-week period, a total of 10 sessions were performed for each rat (Figure 1(b)). The escape latency time and swim speed were measured.

#### 2.4.2. Probe Trial

The probe trial was performed 24 hours after the final acquisition trial (Figure 1(b)). During the probe trial, the platform was removed, and the rats were allowed to swim freely for 120 seconds. The distance to the target quadrant and the percentage of time spent in the target quadrant were measured.

### 2.5. Immunohistochemistry

Immunohistochemical analysis was performed to detect choline acetyltransferase (ChAT) activity. The rats were placed under deep anesthesia using 25% urethane (Sigma, St. Louis, MO) and were perfused through the heart with 200 mL of normal saline (0.9%), followed by 300 mL of 4% formalin (per rat) in 0.1 M phosphate-buffered saline (PBS). The brain was removed, and following 2 hours of postfixation, it was cryoprotected overnight at 4°C using 30% sucrose in 0.1 M PBS. A cryostat (Cool Ace series CA-1500, Eyela, Tokyo, Japan) was used to produce 30 *μ*m thick coronal sections of the hippocampus. The hippocampal slices were washed several times with 0.1 M PBS, and they were then placed on glass slides, dried at 37°C, and stored in a refrigerator. Primary sheep ChAT antibody (1 : 500, monoclonal, Millipore, Billerica, MA) was used to immunostain the slices for ChAT expression. The primary antibody was prepared by diluting the original solution 500-fold in 0.1 M PBS with 0.1% sodium azide (Sigma) buffer. The slices were soaked in the primary antibody at 4°C for 24 hours. They were then washed at least thrice with 0.1 M PBS and were treated with biotinylated universal secondary antibody (Quick Kit; Vector Laboratories, Burlingame, CA) at 37°C for 30 minutes. The slices were again washed at least thrice with 0.1 M PBS and were then soaked in streptavidin peroxidase preformed complex (Quick Kit; Vector Laboratories) at 37°C for 30 minutes. The slices were again washed at least thrice in 0.1 M PBS and were then incubated with diaminobenzidine (DAB; Sigma) for 1 min. Finally, the tissues were washed in 0.1 M PBS and briefly rinsed in distilled water. After dehydrating the tissues, the stained tissues were observed at 40x magnification using a light microscope (Eclipse 80i, Nikon, Tokyo, Japan). The ChAT density in the hippocampus was measured using the Scion image program (Scion Corp., Frederick, MD).

### 2.6. Total RNA Isolation and RT-PCR

For RNA isolation, the hippocampus was dissected out from each group. After decapitation, the brain was removed as quickly as possible and stored at −80°C until use. The tissue of the left hippocampus was homogenized in 800 *μ*L TRIZOL reagent (Roche Diagnostics GmbH, Mannheim, Germany). Then, 200 *μ*L of chloroform (Sigma) was added and mixed well by shaking for 15 seconds. The mixture was allowed to rest for 15 minutes at 24 ± 1°C. The mixture was then centrifuged at 14,000 rpm for 15 minutes at 4°C. The supernatant was collected; 500 *μ*L of isopropanol (Sigma) was added to the supernatant, and the mixture was allowed to rest for 5 minutes at 24 ± 1°C. This mixture was then centrifuged at 14,000 rpm for 8 minutes at 4°C, and the RNA pellet was collected. The RNA pellet was mixed with refrigerated 70% ethanol and DEPC. This mixture was then centrifuged at 7,500 rpm for 5 minutes at 4°C, and the liquid was removed, leaving only the pellet. The remaining ethanol was dried for 5 minutes at 24 ± 1°C. The pellet was dissolving in DEPC-treated water, and the optical density was measured using a spectrophotometer (Biophotometer, Eppendorf, Hamburg, Germany) in order to determine the purity and concentration of RNA. cDNA was synthesized using the total RNA with reverse transcriptase (Bioneer, Daejeon, Korea). The mRNA expression levels of* Creb, Bdnf, Bcl-2, and Bax* were determined using reverse transcription-polymerase chain reaction (RT-PCR). RT-PCR was performed using a Mastercycler Gradient (Eppendorf) with the following conditions: for glyceraldehyde-3-phosphate dehydrogenase* (Gapdh)*, 28 cycles of denaturation at 95°C for 40 s, annealing at 56°C for 40 s, and extension at 72°C for 90 s; for* Creb*, 27 cycles of denaturation at 95°C for 30 s, annealing at 51°C for 30 s, and extension at 72°C for 30 s; for* Bdnf*, 27 cycles of denaturation at 95°C for 30 s, annealing at 57°C for 30 s, and extension at 72°C for 30 s; for* Bcl-2*, 35 cycles of denaturation at 95°C for 40 s, annealing at 55°C for 40 s, and extension at 72°C for 90 s; and for* Bax*, 35 cycles of denaturation at 95°C for 40 s, annealing at 55°C for 40 s, and extension at 72°C for 90 s. The sequences used in RT-PCR are shown in Table 2. The PCR products were subjected to electrophoresis at 100 V using 0.5x TBE buffer (80 mM Tris-HCL, 80 mM boric acid, 2 mM EDTA, and pH 8.3) in a 1.5% agarose gel containing ethidium bromide (EtBr, 10 mg/mL). After electrophoresis, the gel was imaged using an Image Station (Kodak, Rochester, NY), and the density of each band was analyzed using Alphaease FC StandAlone Software (Alpha Innotech, San Leandro, CA).* Creb, Bdnf, Bcl-2*, and* Bax* mRNA expressions were normalized relative to the* Gapdh* mRNA expression.

### 2.7. Statistical Analysis

All data are presented as means ± standard errors of means (SEMs). The Morris water maze data were analyzed with repeated measures analysis of variance (ANOVA). Statistical significance between the groups was verified using Tukey's post hoc test. The immunohistochemistry and PCR data were analyzed using one-way ANOVA followed by Tukey's post hoc test. All statistical analyses were performed using SPSS Software (version 21.0; IBM Corp., Armonk, NY). A *p* value < 0.05 was considered statistically significant.

## 3. Results

### 3.1. Effects of LA on MCAO-Induced Learning and Memory Deficits

**Figure 3:**
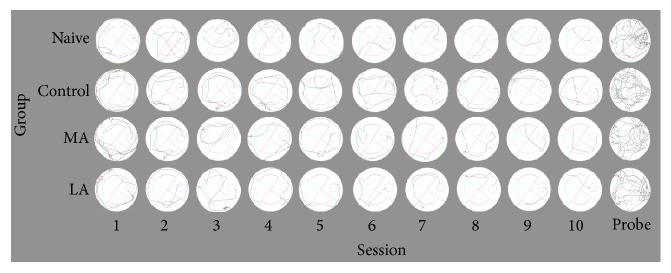
Representative swim paths in the Morris water maze test. The swim paths were analyzed using automated video tracking for all acquisition and probe trials.

**Figure 4:**
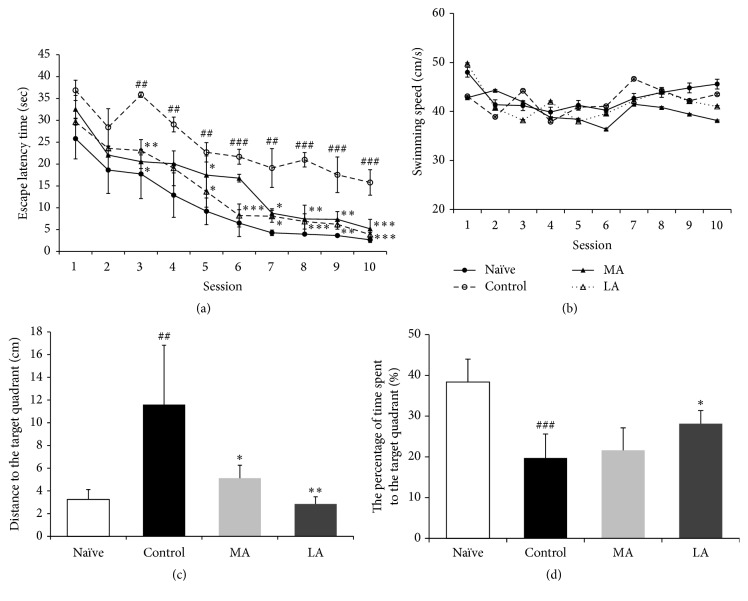
Results of the Morris water maze test. (a) Escape latency time and (b) swim speed during the acquisition trials with a hidden platform. (c) Distance to the target quadrant and (d) percentage of time spent in the target quadrant during the probe trial. Data were analyzed using two-way ANOVA, followed by Tukey's post hoc test. Vertical bars indicate the SEM. Data are expressed as mean ± SEM (*n* = 6 in each group). ^##^*p* < 0.01, and ^###^*p* < 0.001 compared with the naïve group; ^*∗*^*p* < 0.05, ^*∗∗*^*p* < 0.01, and ^*∗∗∗*^*p* < 0.001 compared with the control group.

The representative swim paths in the Morris water maze test are presented in Figure 3. In the acquisition trials, all groups showed a gradual decrease in the escape latency to reach the hidden platform as the number of sessions increased (Figure 4(a)). The escape latency time was significantly lower in the naïve group than in the control group (*p* < 0.01 for sessions 3–5 and 7; *p* < 0.001 for sessions 6 and 8–10). The delayed escape latency time in the LA group showed significant improvement when compared with the delayed escape latency time in the control group (*p* < 0.05 for sessions 3, 5, and 7; *p* < 0.01 for session 9; and *p* < 0.001 for sessions 6, 8, and 10). Additionally, the delayed escape latency time in the MA group showed significant improvement when compared with the delayed escape latency time in the control group (*p* < 0.05 for sessions 6 and 7; *p* < 0.01 for sessions 3, 8, and 9; and *p* < 0.001 for session 10). The delayed escape latency time was not significantly different between the LA and MA groups (*p* = 0.128). Additionally, there was no between-group difference in the swim speed (*p* = 0.147, Figure 4(b)). In the probe trial, the distance to the target quadrant (*F*(3,20) = 8.806, *p* < 0.01; Figure 4(c)) and the time spent in the target quadrant (*F*(3,20) = 13.355, *p* < 0.001; Figure 4(d)) showed significant between-group differences. The distance to the target quadrant was significantly higher and the time spent in the target quadrant was significantly lower in the control group than in the naïve group (*p* < 0.01 and *p* < 0.001, resp.). The distance to the target quadrant was significantly lower in the LA and MA groups than in the control group (MA group: *p* < 0.05; LA group: *p* < 0.01; Figure 4(c)). However, the time spent in the target quadrant was higher in only the LA group than in the control group (*p* < 0.05, Figure 4(d)).

### 3.2. Effects of LA on MCAO-Induced ChAT Immunoreactivity Reduction

**Figure 5:**
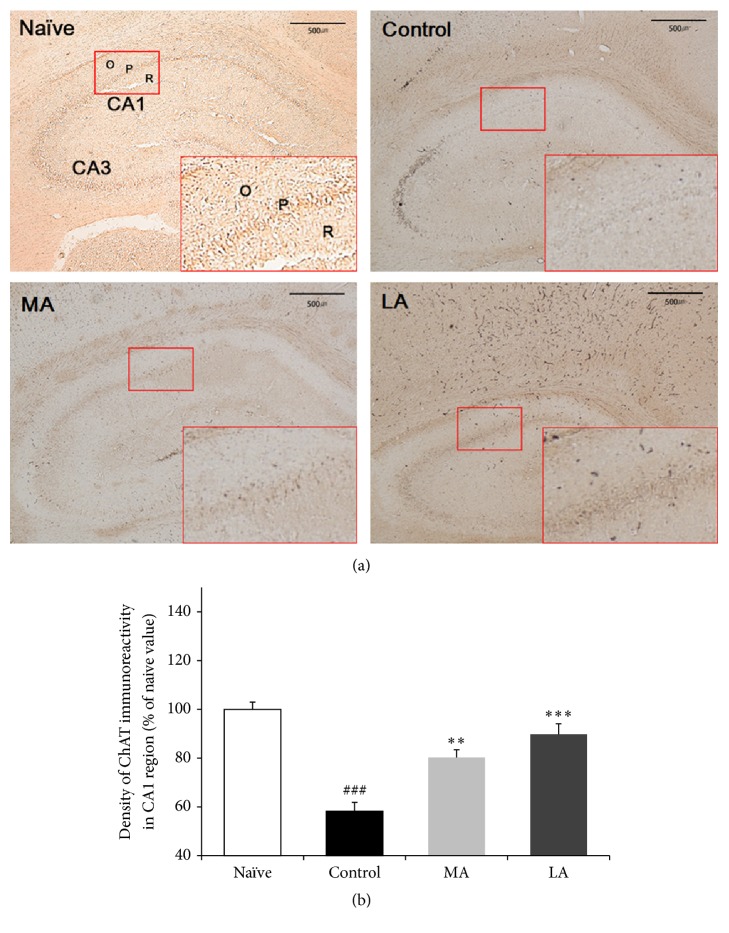
Results of choline acetyltransferase (ChAT) analysis. (a) Representative images showing the density of ChAT in the hippocampus. Red box: CA1 region; O: stratum oriens; P: stratum pyramidale; and R: stratum radiatum. The scale bar represents 500 *μ*m. (b) Percentage (±SE) values for ChAT immunoreactivity in the hippocampal CA1 region after the Morris water maze task. Immunohistochemical data were analyzed using one-way ANOVA, followed by Tukey's post hoc test (*n* = 3 in each group). Vertical bars indicate the SEM. ^###^*p* < 0.001 compared with the naïve group; ^*∗∗*^*p* < 0.01 and ^*∗∗∗*^*p* < 0.001 compared with the control group.

After the behavior test, immunohistochemical analysis was performed to verify cholinergic neuronal cell loss in the rat brains with induced MCAO (Figure 5(a)). The results of ChAT analysis showed that the ChAT density in the hippocampal CA1 region was significantly lower in the control group than in the naïve group (40.23 ± 3.40 (62.85 ± 5.29%) versus 64.29 ± 1.92 (100.0 ± 2.99%), *p* < 0.001; Figure 5(b)). When the ChAT density was compared using one-way ANOVA, we noted significant between-group differences (*F*(3,8) = 44.652, *p* < 0.001). LA and MA treatment significantly reversed the MCAO-induced decrease in ChAT density in the hippocampal CA1 region (57.72 ± 2.77 (89.79 ± 4.31%), *p* < 0.001 and 50.53 ± 3.89 (78.59 ± 6.05%), *p* < 0.01, resp.).

### 3.3. Effects of LA on MCAO-Induced CREB and CRE-Mediated Gene Expressions

**Figure 6:**
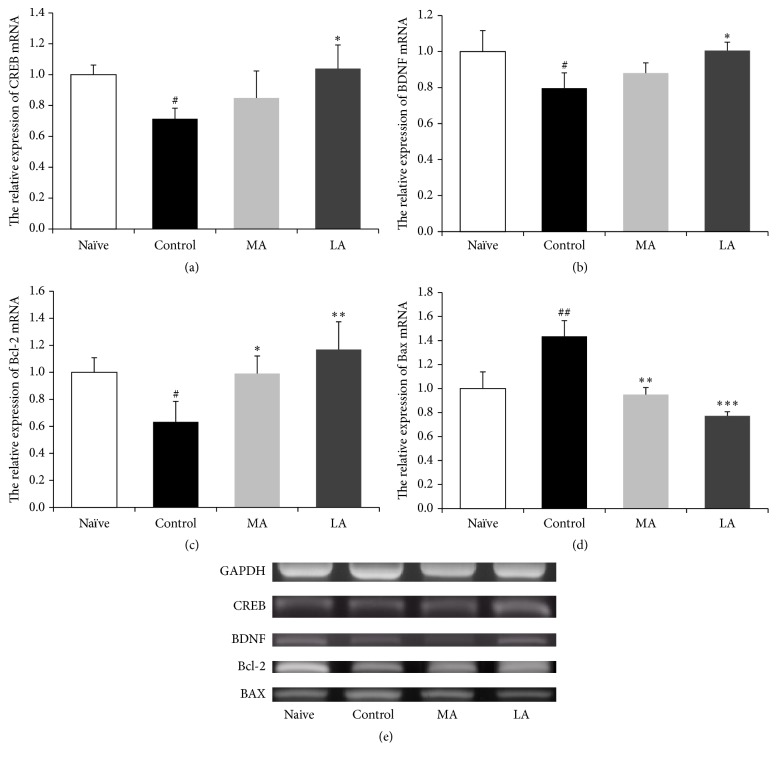
Gene expressions in the hippocampal CA1 region. RT-PCR is used to measure the fold change in the gene expressions of (a) cAMP response element-binding protein (CREB), (b) brain-derived neurotrophic factor (BDNF), (c) B-cell lymphoma 2 (Bcl-2), and (d) Bcl-2-associated X protein (Bax). The mRNA levels are normalized relative to the Gapdh mRNA level. RT-PCR data were analyzed using one-way ANOVA, followed by Tukey's post hoc test (*n* = 3 in each group). Vertical bars indicate SEM. ^#^*p* < 0.05 and ^##^*p* < 0.01 compared with the naïve group; ^*∗*^*p* < 0.05, ^*∗∗*^*p* < 0.01, and ^*∗∗∗*^*p* < 0.001 compared with the control group.

RT-PCR was performed to investigate the effects of LA on the mRNA expressions of* Creb, Bdnf, Bcl-2*, and* Bax* in the hippocampal CA1 region of MCAO rats (Figure 6). The mRNA expressions of* Creb *(*F*(3,8) = 5.651, *p* < 0.05; Figure 6(a)),* Bdnf *(*F*(3,8) = 6.165, *p* < 0.05; Figure 6(b)),* Bcl-2 *(*F*(3,8) = 8.632, *p* < 0.05; Figure 6(c)), and* Bax *(*F*(3,8) = 31.011, *p* < 0.001; Figure 6(d)) showed significant between-group differences. The* Creb*,* Bdnf*, and* Bcl-2* gene expressions showed significant downregulation by approximately 0.7-, 0.8-, and 0.6-fold and the expression of* Bax* showed a significant upregulation by 1.4-fold in the control group when compared with the corresponding expressions in the naïve group (*p* < 0.05, *p* < 0.05, *p* < 0.05, and *p* < 0.01, resp.). The* Creb*,* Bdnf*, and* Bcl-2 *gene expressions were significantly higher and the expression of* Bax* was significantly lower in the LA group than in the control group (*p* < 0.05, *p* < 0.05, *p* < 0.01, and *p* < 0.001, resp.; Figure 6). Conversely, the* Bcl-2 *gene expression was significantly higher and the expression of* Bax* was significantly lower in the MA group than in the control group (*p* < 0.05 and *p* < 0.01, resp.).

## 4. Discussion

Acupuncture is a simple, flexible method in traditional medicine, with few adverse effects [23]. It has recently received attention for its ability to alleviate cognitive deficits and its neuroprotective effects after brain ischemia [2, 3]. LA is a new acupuncture technique using the concept of low-level laser therapy (LLLT), which stimulates acupuncture points based on traditional meridian theory [6, 24]. Because LLLT uses a low irradiation level laser and there is no risk of excessive heat, its mechanism is believed to be associated with a photochemical effect, rather than a thermal effect [6]. Previous studies have reported that LA improves cognitive deficits and memory impairment in various neurological disorders, such as depression [25], autism [9], and Parkinson's disease [8].

In the present study, invasive LA was used to stimulate the acupuncture points GV20 and HT7 for 5 minutes at a wavelength of 650 nm and a power of 30 mW. In conventional noninvasive LA, the acupuncture points are only irradiated with a light source, and, therefore, the procedure has the advantage of being needle- and pain-free [6, 24]. However, it is difficult to make an objective evaluation about the efficacy of LA itself, as laser energy transmission is restricted by the structural characteristics of the skin [26, 27]. Furthermore, noninvasive, low-intensity LA irradiation cannot produce activation of mechanical signal transduction pathways, which results from reorganization of the collagen by acupuncture needles, and it has not been clearly demonstrated whether the photon-mediated effects of LA itself have the same signal transduction pathways as the needle-mediated mechanical effects [28, 29]. In previous studies, stimulation of the acupuncture points HT9 and LR1 with invasive LA at 658 nm in MCAO rats produced antiapoptotic and neuroprotective effects that were similar to the effects with MA, while the efficacy of LA was found to be superior [17]. In LLLT, red and infrared wavelengths of 600–1300 nm and a power of 1–100 mW are the usual stimulation parameters applied for laser acupuncture because there is little absorption by tissues, penetration is excellent, and there is no major damage to tissues with these parameters [26, 30, 31]. Previous studies have reported on the safety of repeated LA treatments in rats (wavelength, 650 nm; maximum power, 60 mW; 5 minutes of stimulation once every 2 days for 16 days) [32].

In the traditional acupuncture theory, the points GV20 and HT7 have been used for a long time in neurological and psychiatric disorders, such as insomnia, epilepsy, and amnesia [33]. A meta-analysis concluded that acupuncture stimulation at the point GV20 reduced the area of infarction in animal models of experimental ischemic stroke, improved neurological function scores, and showed a potential neuroprotective effect [18]. A recent study reported that acupuncture stimulation at the point HT7 in a rat model with cognitive deficit reduced damage to cholinergic neurons and showed a neuroprotective effect by regulating* Creb* and* Bdnf* gene expressions [19].

The Morris water maze is a behavioral instrument used to evaluate cognitive function by measuring spatial learning and memory in rats, based on a logical experimental design [22]. After the spatial working memory information obtained during acquisition trials has been encoded, the spatial reference memory information is retrieved in the subsequent probe trial [34]. The performances in both types of trials were significantly lower in the MCAO rats than in the naïve rats (Figures 4(a), 4(c), and 4(d)); however, there was no significant between-group difference in the mean swim speed (Figure 4(b)). This indicates that MCAO did not cause a motor function deficit in the rat model [35] but rather caused spatial learning and memory impairments due to cognitive function deficits [36]. The reduced working memory and reference memory were both significantly normalized with LA treatment (Figures 4(a), 4(c), and 4(d)). These results indicate that LA can improve impaired hippocampal-dependent learning and memory in MCAO rats.

Brain regions that are supplied by the middle cerebral artery, such as the parietal cortex, hippocampus, and striatum, show severe neural injury after occlusion-induced cerebral ischemia [20]. The hippocampal CA1 region, which is involved in learning, memory, and cognitive function, is especially vulnerable to ischemic insult [11]. Cholinergic neurons originating in the medial septum project to the cortex and hippocampus, and this plays an important role in acetylcholine-related cognitive function [37]. Degeneration of cholinergic innervation is one of the causes of memory decay [38], and a previous study reported a cholinergic deficit in vascular dementia patients [39]. ChAT is involved in the synthesis, storage, and release of acetylcholine and is therefore used as a marker for cholinergic neurons in the hippocampal CA1 region [37]. In the present study, MCAO reduced ChAT immunoreactivity in the CA1 region (Figure 5), indicating that the cholinergic input from the medial septum to the hippocampus was damaged after ischemia [40]. LA significantly restored the decreased ChAT density in the CA1 region (Figure 5), suggesting that it has a beneficial effect on the cognition-associated cholinergic system.

We confirmed that LA treatment in the MCAO-induced cerebral ischemia model rat improved spatial learning and memory and restored ChAT activity in the CA1 region. It has been previously reported that after cerebral ischemia, CREB acts as an important contributor to the survival of neurons by increasing the expression of CRE-mediated genes, including* Bdnf* and* Bcl-2* [14–16]. In the present study, MCAO downregulated the expressions of* Creb* and* Bdnf* in the hippocampal CA1 region in the model rats; however, the expressions were significantly normalized with LA stimulation (Figures 6(a) and 6(b)). In a previous study, increased CREB activity reduced neuronal cell loss in the hippocampal CA1 region, which is vulnerable to ischemic insult, suggesting that ischemic brain injury has an effect on the CRE-mediated transcription system [41]. BDNF is a downstream neuroprotective target of CREB that is involved in neuronal survival after ischemia [14], and it is a neurotrophic factor that can phosphorylate CREB [42]. Previous studies have indicated that the positive-feedback loop between CREB and BDNF could become active in several neuronal populations in response to ischemic brain injury [14, 42]. Additionally, BDNF has been reported to inhibit caspase-3 activity and increase the expression of the* Bcl-2* gene, preventing apoptotic cell death, and thereby reducing ischemic brain injury [15].

The antiapoptotic protein Bcl-2 is another CRE-mediated protein that contributes to neuronal survival [14]. Programmed cell death (PCD) is a factor in delayed neuronal death that occurs after ischemia; however, such apoptotic processes are regulated by the expression ratio of the* Bcl-2* gene and the proapoptotic* Bax* gene [43, 44]. In our study, MCAO reduced* Bcl-2* gene expression and increased* Bax* gene expression in the CA1 region of the rat model (Figures 6(c) and 6(d)). This Bcl-2 dysfunction has been reported to exacerbate ischemic neuronal injury [45]. LA stimulation significantly normalized this imbalance, suggesting that its antiapoptotic effect in the hippocampal CA1 region was the result of significant counterregulation of* Bcl-2* and* Bax* gene expressions [17]. Our results indicate that LA can induce upregulation of* Creb* and CRE-mediated gene expressions and exert a neuroprotective effect in the hippocampal CA1 region.

In the present study, we determined the effects of LA on cognitive impairment in MCAO rats and compared the effects with those of MA. The mechanisms associated with the effectiveness of LA in the treatment of cognitive improvement are unclear. On the other hand, MA has been reported to reduce cognitive decline by stimulating cellular signaling via the needle-mediated mechanical effect, regulating apoptosis, reducing oxidative stress, stabilizing energy metabolism, and restoring synaptic transmission [46]. Our study found that MA stimulation at the points GV20 and HT7 in MCAO rats significantly improved learning and memory deficits in the behavioral test, significantly restored ChAT activity in the hippocampal CA1 region and counterregulated* Bcl-2* and* Bax* gene expressions. Although* Creb* and* Bdnf* gene expressions increased with MA, the expressions were not significantly higher than the expressions in the impaired rats. Considering that several previous studies have shown the regulation of CREB and BDNF neurotrophic signaling by MA stimulation of the points GV20 [47] and HT7 [19], the limited upregulation of* Creb* and* Bdnf* gene expressions shown by MA after cognitive impairment in this study was unexpected. One possible explanation for this discrepancy is the difference in the methods used to induce cognitive impairment. While cognitive impairment was induced via MCAO surgery in the present study, in the previous studies, cognitive impairment was induced via repeated administration of either corticosterone [19] or scopolamine [47]. The model-dependent effects of MA on cognitive deficit cannot be completely explained, and further studies are required to elucidate the differences in the effects of MA in diverse cognitive deficit models.

## 5. Conclusion

In conclusion, the present study confirmed the effects of LA on MCAO with behavioral test in Morris water maze test, ChAT immunoreactivity in the hippocampal CA1 region, and* Creb*,* Bdnf*,* Bcl-2*, and* Bax* gene expressions. It suggests that LA treatment could improve cognitive impairment in MCAO rats to enhance the cholinergic system in the hippocampal CA1 region and to exert a neuroprotective effect by regulating* Creb*,* Bdnf*,* Bcl-2*, and* Bax* gene expressions. The present findings provide evidence for the therapeutic effects of LA on MCAO-induced cognitive impairment. In order to develop treatment standards for the clinical application of LA, subsequent studies should be required for revealing more precisely how these effects of LA depend on acupuncture points and treatment parameters, such as wavelength, power output, frequency, exposure time, and beam profile.

## Figures and Tables

**Table 1:** Specifications of the laser acupuncture system.

Property	Specification	
		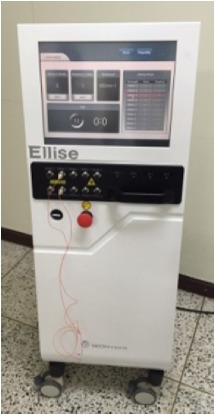
	
	
	
	
Irradiation type	Diode laser	
Wavelength	532 nm, 650 nm, 830 nm, 905 nm, 1064 nm	
Operating current	Each wavelength up to 30 mW	
Core/cladding diameter	50/125 *µ*m	
Pulse duration	1 min; max, 99 min	
Repetition rate	1 Hz; max, 200 Hz	
	
	
	
	
	

**Table 2:** PCR primer sequences.

Gene	Primer sequence (forward and reverse)	Product size (base pair)	Annealing temperature (°C)
*Gapdh*	5′-TGCATCCTGCACCACCAACT-3′	349	56
	5′-CGCCTGCTTCACCACCTTG-3′		
*Creb*	5′-TACCCAGGGAGGAGCAATAC-3′	183	51
	5′-GAGGCAGCTTGAACAACAAC-3′		
*Bdnf*	5′-CAGGGGCATAGACAAAAG-3′	153	57
	5′-CTTCCCCTTTTAATGGTC-3′		
*Bcl-2*	5′-TTGTGGCCTTCTTTGAGTTCGGTG-3′	168	55
	5′-GGTGCCGGTTCAGGTACTCAGTCA-3′		
*Box*	5′-CCTGTGCACCAAGGTGCCGGAACT-3′	498	55
	5′-CCACCCTGGTCTTGGATCCAGCCC-3′		
